# Supplementary material for: Dietary Intakes Are Associated with HDL-Cholesterol in Survivors of Childhood Acute Lymphoblastic Leukaemia
Source: Nutrients. 2019 Dec 5;11(12):2977. doi: 10.3390/nu11122977 (PMC6950746; doi:10.3390/nu11122977)
Supplement: Supplementary file 1 [file nutrients-11-02977-s001.pdf]

Table S1. Cut-off values for cardiometabolic outcomes

Risk factor	Adults	Children
**BMI**		
Overweight	>25 and <30 kg/m^2	>85th and <97th percentile
Obesity	≥30 kg/m^2	≥97th percentile
**Fasting glucose**		
At risk	≥5.6 and <6.1 mmol/L	≥5.6 and <6.1 mmol/L
Prediabetes	≥6.1 mmol/L	≥6.1 mmol/L
**Triglycerides**		
Borderline	≥1.3 and <1.7 mmol/L	≥1.00 and <1.47 mmol/L
High	≥1.7 mmol/L	≥1.47 mmol/L
**LDL-cholesterol**		
Borderline	≥2.6 and <3.4 mmol/L	≥2.85 and <3.36 mmol/L
High	≥3.4 mmol/L	≥3.36 mmol/L
**HDL-cholesterol**		
Low	<1.03 (men) and <1.3 mmol/L (women)	<1.03 mmol/L

BMI, body mass index; LDL, low-density lipoprotein; HDL, high-density lipoprotein.

Table S2. Classification of food groups

Food groups	Food items
Meat	Chicken (grilled, fried, nuggets, casserole, hamburger), Turkey, Beef (grilled, braised, stew, hamburger, veal, pork (grilled, chops), horse meat, lamb, goat, bison, sausage, wiener, cold cut, giblets and meat pâté.
Fish and seafood	White fish, salmon (fresh, canned, smoked), trout, tuna (fresh, canned), fish cake, sardine, mackerel (canned), pollock, lobster, shrimp (fresh, canned), clams, scallops, squid, oysters, mussels, snails.
Dairy	Milk (skim, 1%, 2%, 3.25%), chocolate milk, cheddar, hard cheese, soft cheese, cream cheese, cottage, ricotta, blue cheese, processed cheese, yogurt (plain, Greek, fruits, drinkable)
Fat	Butter, margarine, oil (olive, canola, coco, soya), mayonnaise, cream, lard, bacon.
Vegetables	Asparagus, avocado, broccoli, carrots, celery, mushrooms, cabbage, Brussels sprout, cauliflower, cucumber, zucchini, squash, germinated soybeans, spinach, lettuce, arugula, canned or frozen mixed vegetables, corn, turnip, onion, leeks, sweet potato, pepper (sweet, hot), green peas, radish and tomato.
Legumes	Baked beans, soybean, lentils, red beans, white bean, chickpea and lima beans.
Fruits	Apricot, pineapple, banana, cherry, lemon, lime, kiwi, strawberries, blueberries, blackberries, raspberries, mango, watermelon, melon, cantaloupe, nectarine, clementine, orange, grapefruit, papaya, peach, pear, apple, prunes, dates, grapes, raisin and canned fruit salad.
Fast food	Pizza, hamburger (beef, grilled chicken, fried chicken), gyro sandwich, poutine, hot dog, corndog, club sandwich, hot chicken, chicken wings and fries

Table S3. Calculation of Estimated Energy Requirement

	Estimate Energy Requirement (EER) [1]
**Age and gender groups**	**Equations**
Girl 9-18 years	135.3-(30.8*age [y] + PA*[(10.0*weight [kg] + (934*height [m])]
Boys 9-18 years	88.5-(61.9*age [y]) + PA*(26.7*weight [kg]) + (934*height [m])] + 25
Women ≥ 18 years	354-(6.91*age [y]) + PA*[(9.36*weight [kg]) + (726*height [m])]
Men ≥ 18 years	662-(9.53*age [y]) + PA*[(15.91*weight [kg]) + (539.6*height [m])]

	Physical activity coefficients for EER equations [1]			
	**Sedentary**	**Low active**	**Active**	**Very active**
	Typical daily living activities (e.g., household tasks, walking to the bus)	Typical daily living activities plus 30-60 minutes of daily moderate activity (e.g., walking at 5-7 km/h	Typical daily living activities plus at least 60 minutes of daily moderate activity	Typical daily living activities plus at least 60 minutes of daily moderate activity plus an additional 60 minutes of vigorous activity or 120 minutes of moderate activity
Boys 3-18 years	1.00	1.13	1.26	1.42
Girls 3-18 years	1.00	1.16	1.31	1.56
Men 19y+	1.00	1.11	1.25	1.48
Women 19y+	1.00	1.12	1.27	1.45

1. Governement of Canada, H.C. Dietary Reference Intakes Tables. Available on line: https://www.canada.ca/en/health-canada/services/food-nutrition/healthy-eating/dietary-reference-intakes/tables.html (accessed on September 9, 2019),
